# MCP1-CCR2 and neuroinflammation in the ALS motor cortex with TDP-43 pathology

**DOI:** 10.1186/s12974-019-1589-y

**Published:** 2019-10-30

**Authors:** Javier H. Jara, Mukesh Gautam, Nuran Kocak, Edward F. Xie, Qinwen Mao, Eileen H. Bigio, P. Hande Özdinler

**Affiliations:** 1 Davee Department of Neurology and Clinical Neurological Sciences, Northwestern University Feinberg School of Medicine, Chicago, USA; 2 Les Turner ALS Center, Chicago, USA; 3 Department of Pathology, Northwestern University, Chicago, USA; 4 Mesulam Center for Cognitive Neurology and Alzheimer’s Disease, Northwestern University Feinberg School of Medicine, Chicago, USA; 5 Robert H. Lurie Comprehensive Cancer Center, Northwestern University, Chicago, IL 60611 USA; 6 Department of Neurology, 303 E Chicago Ave., Ward 10-015, Chicago, IL 60611 USA

**Keywords:** Upper motor neurons, Microglia, MCP1-CCR2 axis, TDP-43

## Abstract

**Background:**

The involvement of non-neuronal cells and the cells of innate immunity has been attributed to the initiation and progression of ALS. TDP-43 pathology is observed in a broad spectrum of ALS cases and is one of the most commonly shared pathologies. The potential involvement of the neuroimmune axis in the motor cortex of ALS patients with TDP-43 pathology needs to be revealed. This information is vital for building effective treatment strategies.

**Methods:**

We investigated the presence of astrogliosis and microgliosis in the motor cortex of ALS patients with TDP-43 pathology. prpTDP-43^A315T^-UeGFP mice, corticospinal motor neuron (CSMN) reporter line with TDP-43 pathology, are utilized to reveal the timing and extent of neuroimmune interactions and the involvement of non-neuronal cells to neurodegeneration. Electron microscopy and immunolabeling techniques are used to mark and monitor cells of interest.

**Results:**

We detected both activated astrocytes and microglia, especially rod-like microglia, in the motor cortex of patients and TDP-43 mouse model. Besides, CCR2+ TMEM119- infiltrating monocytes were detected as they penetrate the brain parenchyma. Interestingly, Betz cells, which normally do not express MCP1, were marked with high levels of MCP1 expression when diseased.

**Conclusions:**

There is an early contribution of a neuroinflammatory response for upper motor neuron (UMN) degeneration with respect to TDP-43 pathology, and MCP1-CCR2 signaling is important for the recognition of diseased upper motor neurons by infiltrating monocytes. The findings are conserved among species and are observed in both ALS and ALS-FTLD patients.

## Background

Amyotrophic lateral sclerosis (ALS), one of the most complex neurodegenerative diseases, is characterized by progressive degeneration of cortical and spinal components of motor neuron circuitry [1, 2]. The importance of cortex for the initiation and modulation of voluntary movement is well-established [3], and revealing the contribution of the diseased cortex to ALS pathology is of great importance [4]. The upper motor neurons (UMN), known as Betz cells in humans and corticospinal motor neurons (CSMN) in mice, are one of the critical players of motor function as they initiate and modulate voluntary movement [5].

Even though ALS manifests itself with motor neuron degeneration, the contribution of cells that initiate and modulate immune response cannot be overlooked. Astrogliosis and innate immune response of tissue-resident microglia has been described in patients and mouse models of ALS [6–9]. However, these studies focused mostly on the spinal cord. Our previous analyses revealed the presence of microgliosis in the diseased motor cortex and interaction of activated microglia with degenerating Betz cells of familial and sporadic ALS patients [10], findings recapitulated in the motor cortex of hSOD1^G93A^ mice [11, 12].

To investigate the potential involvement of neuroinflammation especially in the motor cortex of ALS, we focused our attention to TDP-43 pathology, the most common pathology detected in sporadic and familial cases of ALS as well as ALS patients with FTLD (frontotemporal lobar degeneration) [9]. Cytoplasmic accumulation of phosphorylated TDP-43 protein in part defines TDP-43 proteinopathy, and it occurs even in the absence of mutations in the *TARDBP* gene. Interestingly, one of the mouse models generated to investigate the underlying causes of TDP-43 mediated neurodegeneration, the prpTDP-43^A315T^ mice, recapitulated many aspects of the human pathology [13], and the cellular events that contribute to CSMN degeneration were identical to the cellular events that are responsible for Betz cell vulnerability and degeneration in ALS patients with TDP-43 pathology [14]. We thus investigated the potential involvement of immune response in the motor cortex of ALS patients with TDP-43 pathology as well as ALS-FTLD patients with proven TDP-43 inclusions. Additionally, we crossed prpTDP-43^A315T^ with UCHL1-eGFP mice to mark CSMN with eGFP expression in the prpTDP-43^A315T^ background, to bring cellular clarity to our motor cortex investigations and to visualize CSMN with respect to other non-neuronal cells.

Our findings reveal the involvement of astrogliosis and microgliosis especially in layer 5 of the motor cortex in ALS and ALS-FTLD patients and in mice. We report the presence of CCR2+ infiltrating monocytes penetrating the brain parenchyma and diseased Betz cells expressing MCP1, the chemoattractant ligand for CCR2. These results mark a common pathology shared among one of the broadest spectrum of ALS patients and conserved among two distinct species, further suggesting its essential contribution to disease pathology in ALS cortex.

## Materials and methods

### Postmortem human brain samples

Postmortem human tissue was collected according to protocols approved by Northwestern University’s Institutional Review Board. Clinical records were available for every patient. Neuropathologists with expertise in neurodegenerative disorders examined all samples. Brains were fixed as reported [14]. Areas of the primary motor cortex (Brodmann area 4) were retrieved and processed as reported [14]. This study includes motor cortex isolated from normal control cases with no neurologic disease (*n* = 3), ALS patients with cortex involvement and TDP-43 pathology (*n* = 9), and ALS-FTLD patients with TDP-43 pathology (*n* = 3; Table 1). Presence of TDP-43 pathology was confirmed by pS409/410–2 (1:5000 dilution; AEC chromogen, Cosmo-Bio USA, Carlsbad, CA, USA) [15].

**Table 1:** The table of postmortem human samples used in this study

Gender	Age of onset	Age of death	Clinical diagnosis and pathological assessment	TDP + NCIs	TDP + GCIs	TDP + DNs	PMI/h
M	59	61	sALS with TDP-43	+	0	0	29
M	53	61	sALS with TDP-43	+	0	0	13
M	71	73	sALS with TDP-43	0	+	0	23
M	61	64	sALS with TDP-43	0	+	0	14
M	59	64	sALS with TDP-43	+	++	+	19
M	37	40	sALS with TDP-43	+	++	+	19
M	55	57	fALS with TDP-43	+	+++	0	18
F	78	82	fALS with TDP-43	++	0	0	36
F	62	64	sALS with TDP-43	+	+	0	19
F	NA	55	ALS FTLD TDP type B	+	++	0	5
F	NA	70	ALS FTLD TDP type B	+	+++	+	11
M	NA	62	ALS FTLD TDP type B	Rare	+	Rare	12
M	N/A	54	Normal control	0	0	0	12
M	N/A	72	Normal control	0	0	0	14
F	N/A	78	Normal control	0	0	0	8

Information about age, sex, clinical diagnosis, and the type of TDP-43 pathology is included. *NCIs* neuronal cytoplasmic inclusions, *GCIs* glial/microglial cytoplasmic inclusions, *DNs* extracellular dystrophic neuritis, *PMI/h* postmortem interval in hours

### Mice

All animal experiments followed the standards set by National Institutes of Health and were performed in accordance to animal protocols approved by the Northwestern University Animal Care and Use committee. Mice were on a C57/BL6 background. WT, prpTDP-43^A315T^ (Jackson Laboratory, stock#. 010700), UCHL1-eGFP (generated by the Ozdinler Lab and made available at Jackson Laboratory, stock#. 022476) [16], and prpTDP-43^A315T^-UeGFP mice (generated by the Ozdinler Lab) are used [14].

### Tissue collection, processing, and immunocytochemistry

Mice were deeply anesthetized and perfused as previously described [11]. The brain was dissected, post-fixed in 4% PFA overnight, stored in PBS with 0.01% sodium azide, and sectioned at 50 μm using Leica vibratome (Leica VT1000S, Leica Inc., Nussloch, Germany). Floating sections were processed for immunocytochemistry [10]. All antibodies were obtained from Abcam (Cambridge, MA, USA) unless otherwise stated. In this study, chicken anti-GFP (1:500), rat anti-GFAP (1:1000; Invitrogen), rabbit anti-Iba1 (1:500; Wako), TMEM 119 (1:500), mouse anti-CCR2 (1:500), and rabbit anti-CD31 (1:500) were used.

For postmortem human samples, slides were baked for 60 min at 60 °C, deparaffinized with xylene for 5 min, and rehydrated in ethanol (100, 95, 70, and 50%). Antigen retrieval was performed as previously reported [17]. In this study, chicken anti-Map 2 (1:200, Abcam, Cambridge, MA, USA), rat anti-GFAP (1:1000; Invitrogen, Thermo Fisher Scientific, Rockford, IL, USA), rabbit anti-Iba1 (1:500; Wako), TMEM 119 (1:1000), mouse anti-CCR2 (1:500), and rabbit anti-CD31 (1:500) were used.

### Immunocytochemistry coupled with electron microscopy (EM)

#### Human

Motor cortex was dissected from PFA-fixed autopsy brain samples, cut into approximately 1-mm cubes, and post-fixed in 2.5% glutaraldehyde for 2 h at RT. Sections were then post-fixed in buffered 2% osmium tetroxide (OsO4) (Electron Microscopy Sciences, Hatfield, PA), rinsed with distilled water and stained in 1% uranyl acetate (Electron Microscopy Sciences, Hatfield, PA), again rinsed with distilled water, dehydrated in ascending grades of ethanol with transition fluid propylene oxide (Electron Microscopy Sciences, Hatfield, PA), and embedded in resin mixture with Embed 812 (Electron Microscopy Sciences, Hatfield, PA) and cured in a 60 °C oven for 3 days. The sections were mounted on resin block and were sectioned on a Leica Ultracut UC6 ultramicrotome (Leica Inc., Nussloch, Germany). Seventy-nanometer-thin sections were collected on 200 mesh copper–palladium grids. Grids were counter stained with 8% radioactive depleted uranyl acetate and 0.2% lead citrate.

#### Mouse

Mice were perfused with EM grade 4% PFA. One hemisphere of the brain was sectioned at 50 μm, coronally on a vibratome (Leica VT1000S, Leica Inc., Nussloch, Germany), and post-fixed in 2.5% glutaraldehyde for 2 h at RT. Immunocytochemistry was performed using anti-Ctip2 antibody (1:500, Thermo Fisher Scientific, Rockford, IL, USA), and biotinylated goat anti-rat IgG (1:500, Vector Laboratories, Burlingame, CA, USA). Samples were processed for prepared for EM as previously reported [14].

### Imaging and data collection

Nikon Eclipse TE2000-E (Nikon Inc., Melville, NY, USA), Leica TCS SP5 confocal microscope (Leica Inc., Bensheim, Germany), and Zeiss 880 confocal microscope (Carl Zeiss microscopy, Jena, Germany) were used to acquire low- and high-magnification images, respectively. EM grids were examined on FEI Tecnai Spirit G2 TEM (FEI company, Hillsboro, OR, USA), and digital images were captured on a FEI Eagle camera. Electron microscopy imaging was performed at the Center for Advanced Microscopy/Nikon Imaging Center (CAM), at the Northwestern University Feinberg School of Medicine, Chicago.

#### Human

Three random images within the motor cortex (Brodmann area 4) were taken using a 10 × objective per subject. All Iba1+ cells (microglia) and GFAP+ cells (astrocyte) that have a nucleus (DAPI+) were counted in each field of image without bias, and their numbers within the same objective field were determined and averaged per subject. The “quiescent/normal” and “activated” states of astrocytes and microglia were defined based on previously established criteria [10, 11, 18]. Their percent distribution of among all microglia and astrocytes was reported. All counts were performed blindly by same person, for consistency. In addition, large pyramidal neurons located in layer 5 of the motor cortex with a diameter larger than 20 μm and that express MCP1and Map 2 were quantified using the same images.

#### Mouse

The numbers of microglia (Iba1+) and astrocytes (GFAP+) were quantified from three comparable sections obtained from the motor cortex of WT-UeGFP and prpTDP-43^A315T^-UeGFP mice at P30, P60, P90, P120, and P150 (*n* = 3). An equivalent area of the motor cortex in three serial sections (at least ∼ 600 μm apart) was imaged with × 10 objective field per mouse that represents motor cortex area based on the Paxinos brain atlas (section 1: plate 21, Bregma 1.18 mm, interaural 4.98 mm; section 2: plate 25, Bregma 0.74 mm, interaural 4.54 mm; and section 3: plate 30, Bregma 0.14 mm, interaural 3.94 mm). All microglia and astrocytes located in layer 2/3 and layer 5 of the motor cortex with visible nucleus were counted by an experimenter blind to the age and genotype of the mice without bias.

EM was used to obtain high-magnification images from 70-nm-thick sections of the motor cortex (human: Brodmann area 4; mouse: three sections spanning the motor cortex, as described above) isolated from WT (*n* = 3) and prpTDP-43^A315T^ mice (*n* = 3). Similarly, tissues from nine ALS patients and three normal controls were used for analyses. The images (*n* = 2/subject) were taken from random 200 mesh grid fields. Cells penetrating from the blood vessels with macrophagic morphology as described previously [19] were noted, and all cells that are within the 20-μm periphery of the blood vessels were counted, without bias.

### Statistical analyses

Investigators collecting the data and performing the quantifications were blind to the genotype and pathology. After all data were collected, investigators were unblinded and statistical analyses were performed using the Prism software (GraphPad Software Inc., La Jolla, CA, USA). D’Agostino and Pearson normality tests were performed on all data sets. Either Student’s *t* test or ANOVA with post hoc Tukey’s multiple comparison test was used to determine statistical differences between the experimental groups depending on the genotype, experimental conditions, and the disease group. Data are shown as mean ± SEM of at least three replicates and are representative of three independent experiments unless otherwise stated and statistically significant differences were taken at *p* < 0.05, and *p* values or adjusted *p* values are reported in the text.

## Results

### Evidence of microgliosis around Betz cells in ALS with TDP-43 pathology

**Fig. 1:**
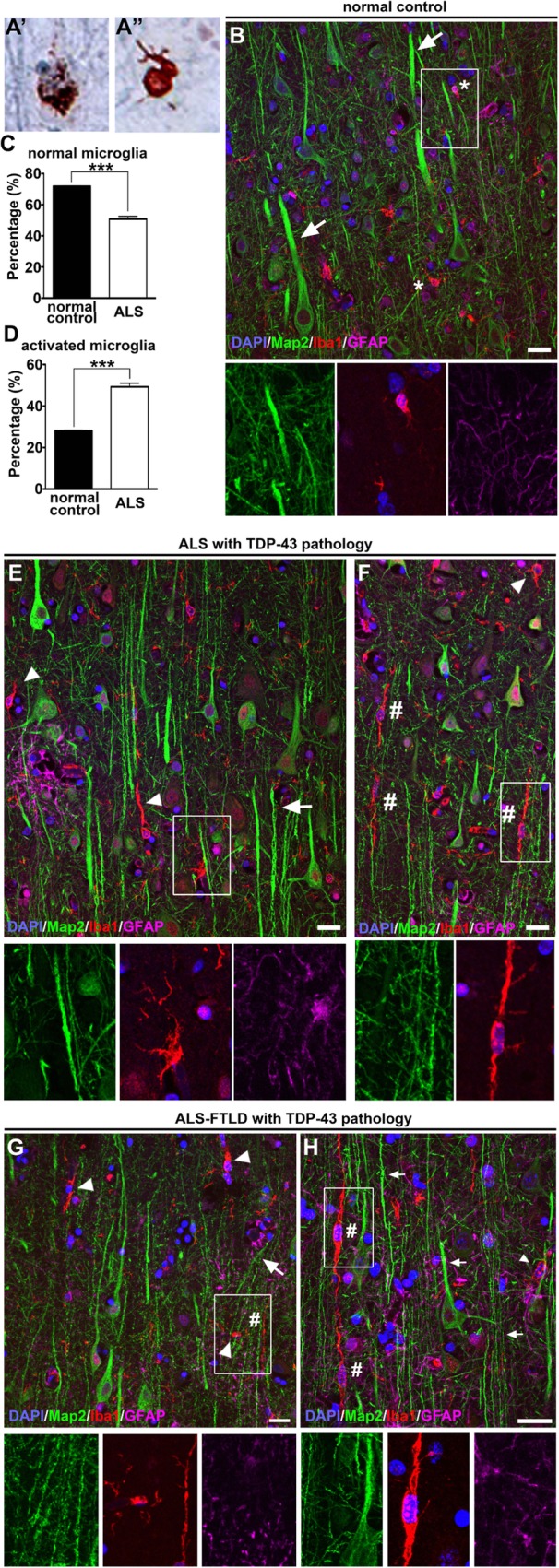
Microgliosis is observed throughout the motor cortex of ALS patients with TDP-43 pathology. Representative images of layer 5 of the motor cortex isolated from postmortem normal controls and ALS patients. Representative image of cortical neuron showing neuronal cytoplasmic inclusions (**a**) and glial cytoplasmic inclusions (**a’**) of pTDP-43 (**b**). Normal controls have large Betz cells (Map 2+) located in layer 5 of the motor cortex with normal apical dendrites (arrows) with no evidence of microgliosis (Iba1+) or astrogliosis (GFAP+). Inset is enlarged bellow. **c** Graph represents percentage of normal microglia per section ± SEM in normal controls (black column) and with TDP-43 pathology (white column). **d** Graph represents percentage of activated microglia per section ± SEM in normal controls (black column) and withTDP-43 pathology (white column). **e**, **f** ALS cases with TDP-43 pathology have smaller Betz cells, microgliosis with rod-like microglia, and astrogliosis. **g**, **h** ALS-FTLD cases with TDP-43 pathology also have smaller Betz cells, microgliosis with rod-like microglia, and astrogliosis. Insets are enlarged bellow. ***p < 0.0001. Asterisk denotes normal microglia, arrows point to apical dendrites, arrowheads point to activated microglia, and number signs point to rod-like microglia. Scale bar = 20 μm

To investigate the role of neuroinflammation with respect to ALS with TDP-43 pathology, we first investigated the presence of microgliosis and astrogliosis in the primary motor cortex Brodmann area 4 of normal controls, ALS cases, and ALS-FTLD cases with TDP-43 pathology (Fig. 1A’, A”; Table 1). Microglia and astrocyte morphology which allows for the differentiation of “quiescent/normal” microglia has a small cell body and ramified thin processes, and “activated” microglia have enlarged cell body and thick and short processes as previously defined by us and others [10, 18]. GFAP signal was low in the normal control brain (Fig. 1B), and the total number of astrocytes were comparable between normal controls (46 ± 1, *n* = 3) and ALS cases (47 ± 2, *n* = 9; *p* = 0.7571). However, the percentage of activated astrocytes increased about 34% in ALS patients with TDP-43 pathology (normal controls: 24 ± 5%, *n* = 3; ALS with TDP-43 pathology: 58 ± 3, *n* = 9, *p* < 0.0001), in contrast to percentage of quiescent astrocytes in patients (normal controls: 76 ± 5%, *n* = 3; ALS with TDP-43 pathology: 41 ± 2, *n* = 9, *p* < 0.0001; Fig. 1E, G). Similar observations were made with microglia. The total number of microglia were comparable between normal controls (36 ± 1, *n* = 3) and ALS cases (36 ± 2, *n* = 9; *p* = 0.786); there was a significant decrease of quiescent microglia in ALS patients with TDP-43 pathology (normal controls: 72 ± 1%, *n* = 3; ALS with TDP-43 pathology: 51 ± 2, *n* = 9, *p* < 0.0001; Fig. 1C) and about 21% increase of activated microglia in ALS patients with TDP-43 pathology (normal controls: 28± 1%, *n* = 3; ALS with TDP-43 pathology: 49 ± 2, *n* = 9, *p* < 0.0001; Fig. 1D). Betz cells with small soma and vacuolated apical dendrite, consistent with characteristics of degenerating Betz cells [17] (arrow), were surrounded by activated microglia (arrowhead). In contrast, “quiescent” microglia (asterisk) were in close proximity to healthy Betz cells in normal controls (Fig. 1B). Interestingly, the presence of unique rod-like microglia characterized by elongated nuclei was observed exclusively in ALS patients (Fig. 1F, H, number sign), and they were found mostly aligned with degenerating apical dendrites.

### Astrogliosis and microgliosis in the motor cortex of prpTDP-43^A315T^-UeGFP mice

**Fig. 2:**
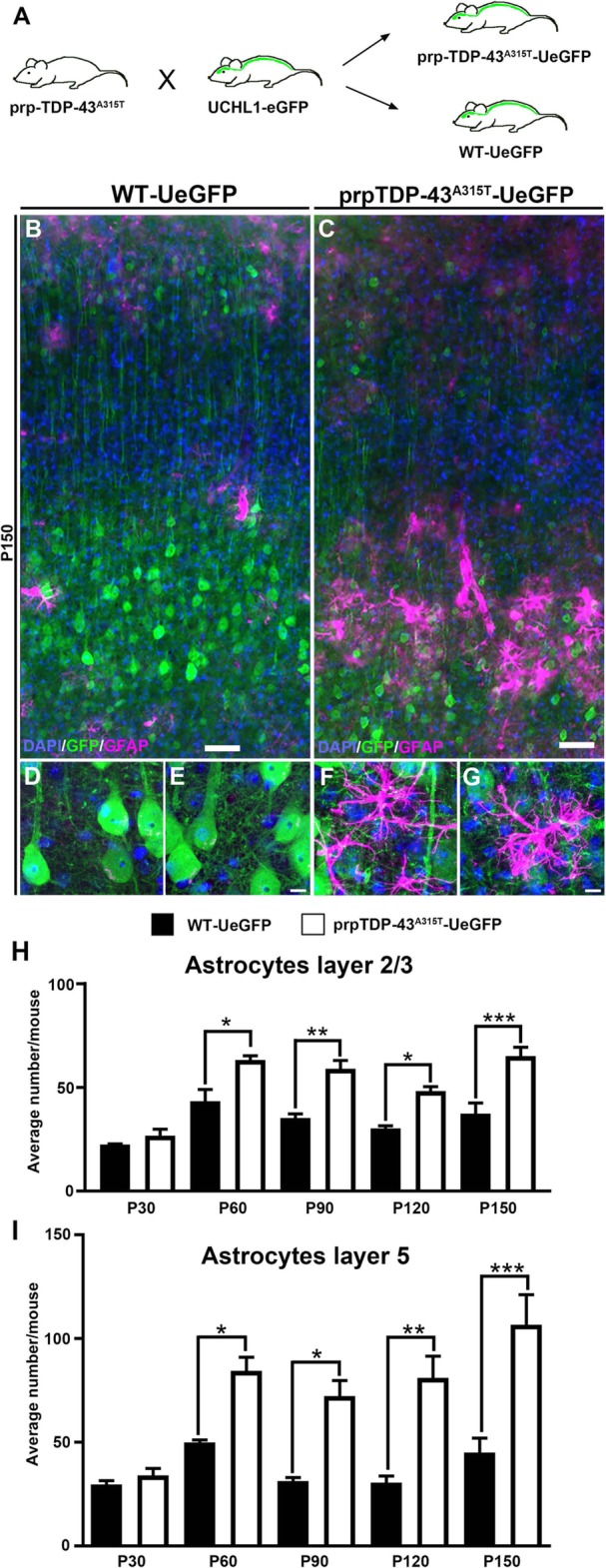
There is increased astrogliosis in the motor cortex of prpTDP-43^A315T^-UeGFP. **a** Breeding strategy to generate prpTDP-43^A315T^-UeGFP mice upon crossbreeding of prpTDP-43^A315T^ and UCHL1-eGFP mice. **b** A representative low-magnification image of motor cortex of WT-UeGFP mice showing presence of few astrocytes with high-magnification images from layer 5 of motor cortex in (**d** and **e**). **c** A representative low image of motor cortex of prpTDP-43^A315T^-UeGFP mice showing presence of increased number of astrocytes with magnification images from layer 5 of motor cortex of prpTDP-43^A315T^-UeGFP mice showing astrocytes in close proximity to corticospinal motor neurons (**f** and **g**). **h** Quantification of number of astrocytes in layer 2/3 and **i** layer 5 shows a progressive increase in astrocytes in the motor cortex. Scale bar: **a**, **d** = 10 μm; **b**, **c**, **e**, **f** = 20 μm. *p < 0.05, **p < 0.001, ***p < 0.0001

We previously generated and characterized prpTDP-43^A315T^-UeGFP mice, a novel TDP-43 mouse model with genetically-labeled eGFP+ CSMN, by crossing the well-characterized prpTDP-43^A315T^ mice with UCHL1-eGFP (Fig. 2a). CSMN of these mice faithfully recapitulate the cellular defects observed in Betz cells of ALS patients with TDP-43 pathology [14]. We investigate the involvement of neuroinflammation in layer 2/3 of the motor cortex, where CSMN receives most of its modulatory inputs, and layer 5 [20], where CSMN soma is located.

Astrocyte numbers were higher in the motor cortex of prpTDP-43^A315T^-UeGFP mice with prominent astrogliosis, especially in the layer 5 (Fig. 2b–g), but numbers were comparable in layer 2/3 at P30 (WT-UeGFP: 23 ± 1 astrocytes; prpTDP-43^A315T^-UeGFP: 27 ± 3 astrocytes, *n* = 3 mice; adjusted *p* = 0.994). However, the presence of astrocytes increased at P60 (WT-UeGFP: 43 ± 5 astrocytes; prpTDP-43^A315T^-UeGFP: 63 ± 5 astrocytes, *n* = 3 mice, adjusted *p* = 0.014), P90 (WT-UeGFP: 35 ± 2 astrocytes; prpTDP-43^A315T^-UeGFP: 59 ± 4 astrocytes, *n* = 3 mice, adjusted *p* = 0.014), P120 (WT-UeGFP: 30 ± 1 astrocytes; prpTDP-43^A315T^-UeGFP: 48 ± 2 astrocytes, *n* = 3 mice, adjusted *p* = 0.004), and P150 (WT-UeGFP: 34 ± 5 astrocytes; prpTDP-43^A315T^-UeGFP*:* 62 ± 4 astrocytes, *n* = 3 mice, adjusted *p* = 0.014) (Fig. 2h). Initially, there was no difference in the numbers of astrocytes in layer 5 of the motor cortex at P30 (WT-UeGFP: 30 ± 2 astrocytes; prpTDP-43^A315T^-UeGFP: 34 ± 1 astrocytes, *n* = 3 mice). However, the number of astrocytes in layer 5 increased at P60 (WT-UeGFP: 50 ± 1 astrocytes; prpTDP-43^A315T^-UeGFP: 84 ± 6 astrocytes, *n* = 3 mice, adjusted *p* = 0.031), P90 (WT-UeGFP: 31 ± 2 astrocytes; prpTDP-43^A315T^-UeGFP: 72 ± 7 astrocytes, *n* = 3 mice, adjusted *p* = 0.027), P120 (WT-UeGFP: 31 ± 3 astrocytes; prpTDP-43^A315T^-UeGFP: 81 ± 11 astrocytes, *n* = 3 mice, adjusted *p* = 0.032), and P150 (WT-UeGFP: 45 ± 7 astrocytes; prpTDP-43^A315T^-UeGFP: 107 ± 14 astrocytes, *n* = 3 mice, adjusted *p* = 0.033) (Fig. 2i).

**Fig. 3:**
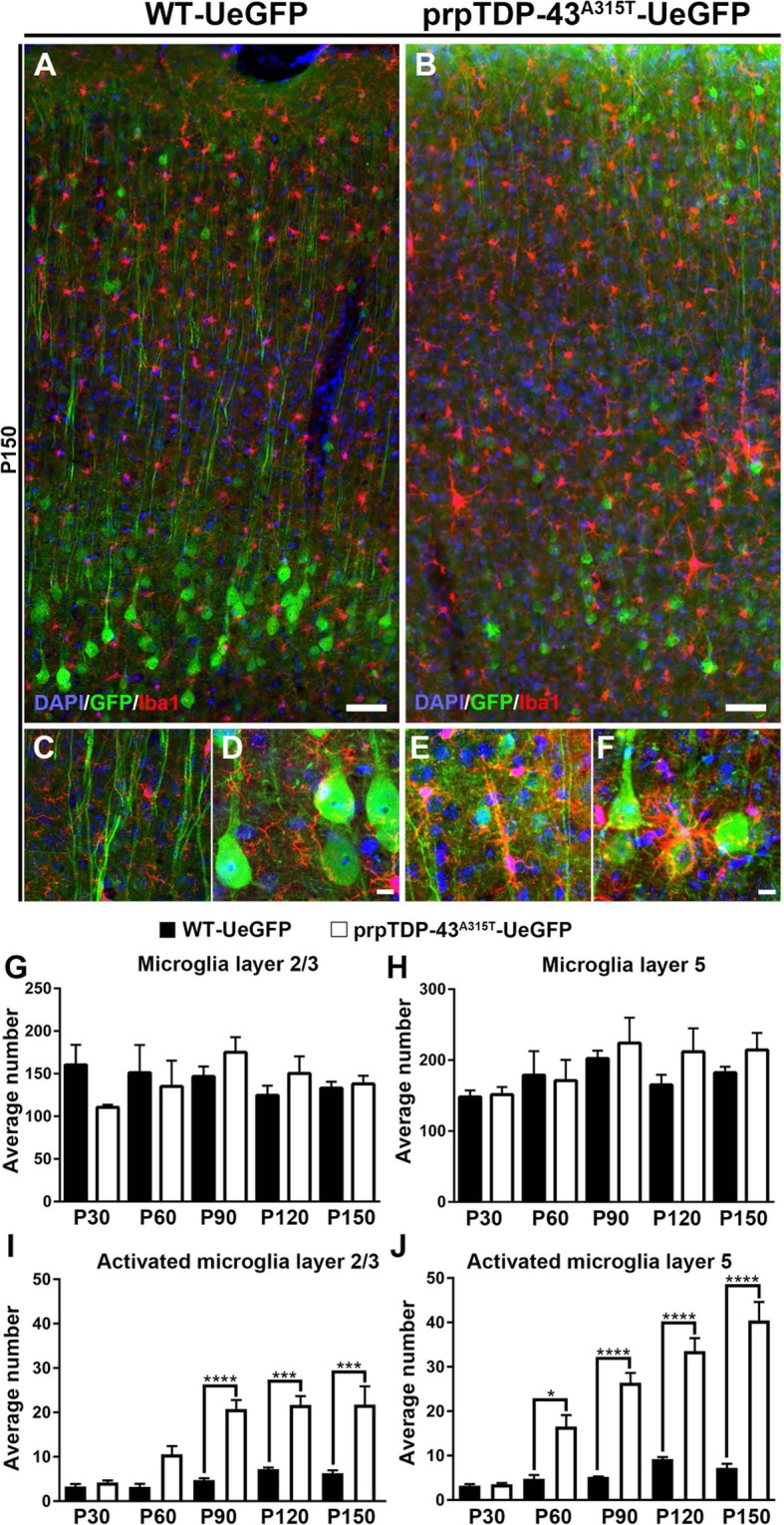
Microgliosis is prominent in the motor cortex of prpTDP-43^A315T^-UeGFP. **a** A representative image of motor cortex of WT-UeGFP mice showing normal presence of microglia (Iba1+) with high-magnification images from layer 2/3 and layer 5 of motor cortex in (**c** and **d**) respectively. **b** A representative image of motor cortex of prpTDP-43^A315T^-UeGFP mice showing microgliosis with high-magnification images from layer 2/3, and layer 5 of motor cortex showing activated microglia adjacent to the apical dendrite and soma of corticospinal motor neurons in (**e** and **f**), respectively. **g**, **h** Quantification of number of microglia in layer 2/3 (**g**) and layer 5 (**h**) shows no difference in number of total microglia the motor cortex. **i**, **j** Quantification of activated microglia in layer 2/3 (**i**) and layer 5 shows a progressive increase of activated microglia in the motor cortex of prpTDP-43^A315T^-UeGFP mice. Scale bar: **a**, **d** = 10 μm; **b**, **c**, **e**, **f** = 20 μm. *p < 0.01, ***p < 0.0004, ****p < 0.0001

We next investigated whether there is microgliosis in layer 2/3 and layer 5 in the motor cortex with respect to disease initiation and/or progression (Fig. 3). The total numbers of microglia did not change over time in layer 2/3 at P30 (WT-UeGFP: 160 ± 23 microglia; prpTDP-43^A315T^-UeGFP: 110 ± 3 microglia, *n* = 3 mice), P60 (WT-UeGFP: 151 ± 32 microglia; prpTDP-43^A315T^-UeGFP: 135 ± 30 microglia, *n* = 3 mice), P90 (WT-UeGFP: 146 ± 11 microglia; prpTDP-43^A315T^-UeGFP: 175 ± 18 microglia, *n* = 3 mice), P120 (WT-UeGFP: 124 ± 11 microglia; prpTDP-43^A315T^-UeGFP: 150 ± 20 microglia, *n* = 3 mice), and P150 (WT-UeGFP: 133 ± 7 microglia; prpTDP-43^A315T^-UeGFP: 138 ± 9 microglia, *n* = 3 mice; Fig. 3g), and in layer 5 at P30 (WT-UeGFP: 148 ± 9 microglia; prpTDP-43^A315T^-UeGFP: 151 ± 10 microglia, *n* = 3 mice), P60 (WT-UeGFP: 178 ± 33 microglia; prpTDP-43^A315T^-UeGFP: 171 ± 29 microglia, *n* = 3 mice), P90 (WT-UeGFP: 202 ± 11 microglia; prpTDP-43^A315T^-UeGFP: 224 ± 35 microglia, *n* = 3 mice), P120 (WT-UeGFP: 165 ± 14 microglia; prpTDP-43^A315T^-UeGFP: 211 ± 32 microglia, *n* = 3 mice), and P150 (WT-UeGFP: 182 ± 8 microglia; prpTDP-43^A315T^-UeGFP: 214 ± 24 microglia, *n* = 3 mice; Fig. 3h). However, activated microglia became prominent with disease progression. In layer 2/3, the levels were comparable at P30 (WT-UeGFP: 3 microglia; prpTDP-43^A315T^-UeGFP: 4 microglia, *n* = 3 mice), but increased at P60 (WT-UeGFP: 3 microglia; prpTDP-43^A315T^-UeGFP: 11 ± 2 microglia, *n* = 3 mice, adjusted *p* = 0.046), P90 (WT-UeGFP: 5 ± 1 microglia; prpTDP-43^A315T^-UeGFP: 21 ± 2 microglia, *n* = 3 mice, adjusted *p* = 0.013), P120 (WT-UeGFP: 7 microglia; prpTDP-43^A315T^-UeGFP: 22 ± 2 microglia, *n* = 3 mice, adjusted *p* = 0.016), and P150 (WT-UeGFP: 6 microglia; prpTDP-43 ^A315T^-UeGFP: 23 ± 3 microglia, *n* = 3 mice, adjusted *p* = 0.042). The presence of activated microglia was more evident in layer 5 and became significant with disease progression. Even though the levels were comparable at P30 (WT-UeGFP: 3 microglia; prpTDP-43^A315T^-UeGFP: 4 microglia, *n* = 3 mice), the extent of microgliosis was increased at P60 (WT-UeGFP: 5 microglia; prpTDP-43^A315T^-UeGFP: 17 ± 2 microglia, *n* = 3 mice, adjusted *p* = 0.034), P90 (WT-UeGFP: 5 ± 1 microglia; prpTDP-43^A315T^-UeGFP: 26 ± 2 microglia, *n* = 3 mice, adjusted *p* = 0.010), P120 (WT-UeGFP: 9 microglia; prpTDP-43^A315T^-UeGFP: 34 ± 3 microglia, *n* = 3 mice, adjusted *p* = 0.012), and P150 (WT-UeGFP: 7 ± 1 microglia; prpTDP-43^A315T^-UeGFP: 33 ± 4 microglia, *n* = 3 mice, *p* = 0.012; Fig. 3j).

### Astrogliosis and microgliosis in the motor cortex of prpTDP-43^A315T^-UeGFP mice are similar to that of ALS patients with TDP-43 pathology

**Fig. 4:**
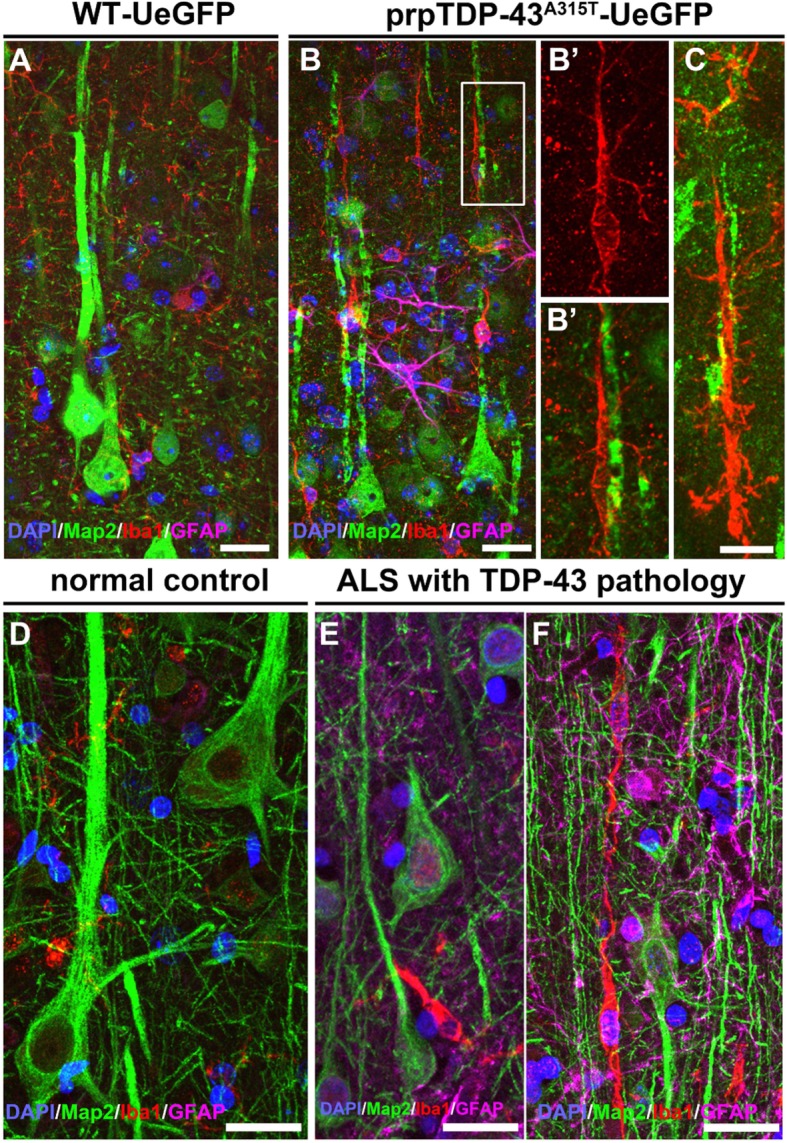
Microgliosis with respect to TDP-43 pathology is conserved among species. **a** A representative image of motor cortex of WT-UeGFP mice with healthy CSMN and normal levels of microglia and astrocytes. **b** A representative image of motor cortex of prpTDP-43^A315T^-UeGFP with activated microglia surrounding degenerating CSMN apical dendrites. Insets enlarged to the right (**b’**). **c** A representative image of rod-like microglia near a degenerating CSMN apical dendrite in the motor cortex of prpTDP-43^A315T^-UeGFP. **d** A representative image of layer 5 of the primary motor cortex (Brodmann area 4) isolated from normal controls showing healthy Betz cells and normal levels of microglia and astrocytes. **e**, **f** A representative image of layer 5 of the primary motor cortex (Brodmann area 4) isolated from postmortem ALS cases with TDP-43 pathology demonstrate presence of smaller Betz cells (Map 2+) surrounded by activated microglia (Iba1+) (**e**), and rod-like microglia near a degenerating apical dendrites of Betz cells are observed with prominent astrogliosis (**f**). Scale bar = 20 μm

The similarities between findings in the motor cortex of the prpTDP-43^A315T^-UeGFP mice and the motor cortex of ALS patients with TDP-43 pathology were remarkable (Fig. 4). The diseased upper motor neurons are near astrocytes and microglia both in mice (Fig. 4B, C) and in patients (Fig. 4E, F). Activated microglia with enlarged cell bodies and thick processes wrapped the degenerating CSMN apical dendrites (Fig. 4B’) and apical dendrites of Betz cells (Fig. 4E, F). Additionally, dysmorphic microglia with characteristics of rod-like microglia were observed both next to the degenerating CSMN (Fig. 4C) and Betz cell apical dendrites (Fig. 4E, F). In contrast, healthy Betz cells were devoid of activated microglia and astrogliosis in their proximity (Fig. 4D). Interestingly, numerous rod-like microglia were noted along the degenerating apical dendrites, further suggesting their potential involvement in UMN degeneration in both species.

### Infiltrating monocytes enter the motor cortex of prpTDP-43^A315T^-UeGFP mice

**Fig. 5:**
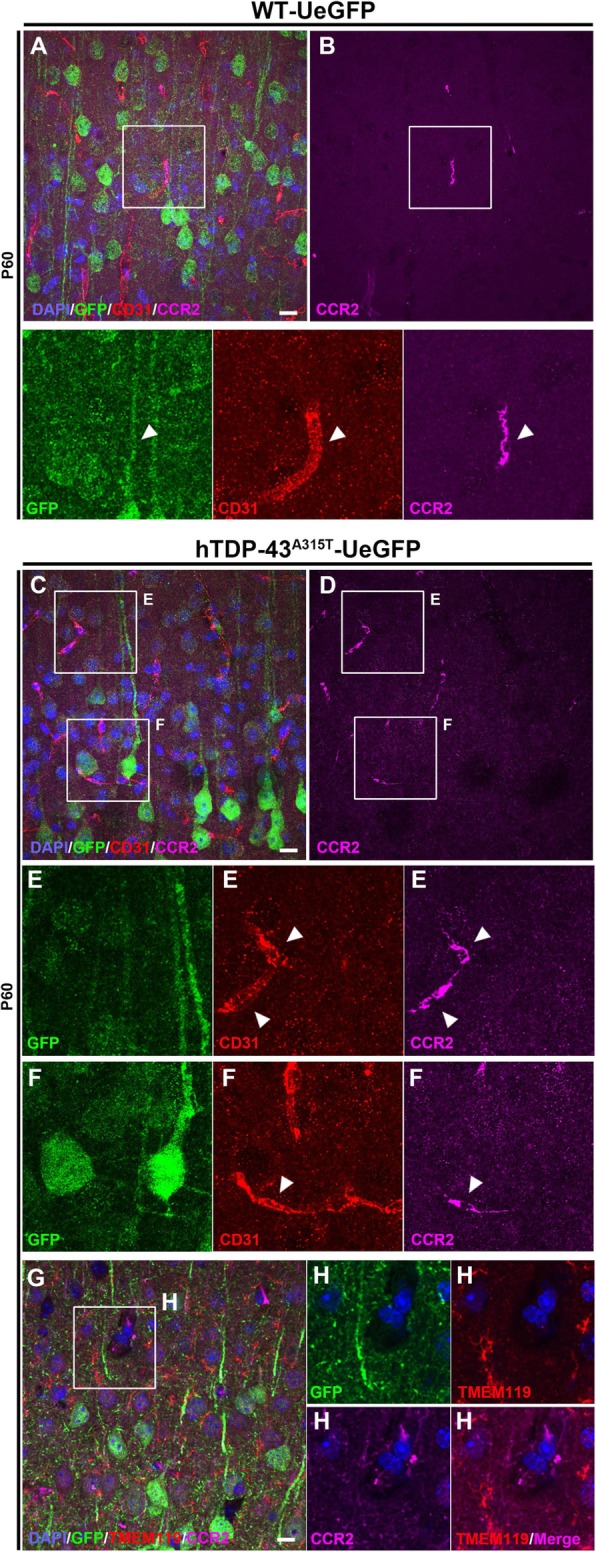
Evidence of infiltrating monocyte contribution in the motor cortex of prpTDP-43^A315T^-UeGFP mice. **a** A representative image of motor cortex of WT-UeGFP mice showing an infiltrating monocyte (CCR2+) within the blood vessel (CD31+), see arrowheads. **c**, **d** A representative image of motor cortex of prpTDP-43^A315T^-UeGFP mice showing higher numbers of infiltrating monocytes (CCR2+) within the blood vessel (CD31+), see arrowheads and insets enlarged bellow in E, F. **g**, **h** A representative image of motor cortex of prpTDP-43^A315T^-UeGFP mice showing infiltrating monocytes (CCR2+) that lack TMEM119 expression within the blood vessel. Inset is enlarged to the right. Scale bar = 20 μm

Upon visualization of microgliosis and astrogliosis surrounding Betz cells and CSMN in the motor cortex, we next asked if peripheral immune cells contribute to UMN degeneration. We previously detected infiltrating monocytes expressing CCR2 in the motor cortex of hSOD1^G93A^ mice, one of the best-characterized mouse models of ALS [10]. Therefore, we investigated whether CCR2+ infiltrating monocytes were also present in the brain parenchyma and whether they enter from the bloodstream. In the WT-UeGFP motor cortex, very few CCR2+ infiltrating monocytes were observed (Fig. 5A, B). In striking contrast, numerous CCR2+ infiltrating monocytes were detected within and around the blood vessels in the motor cortex of prpTDP-43^A315T^-UeGFP mice (Fig. 5C, D and insets E, F). TMEM119 expression profile further demonstrated that CCR2+ cells were indeed infiltrating monocytes and not of microglia origin (Fig. 5G, H). They were in close proximity to CSMN both at the soma and apical dendrite levels. Interestingly, apical dendrites displayed signs of degeneration with vacuolation and disintegration.

**Fig. 6:**
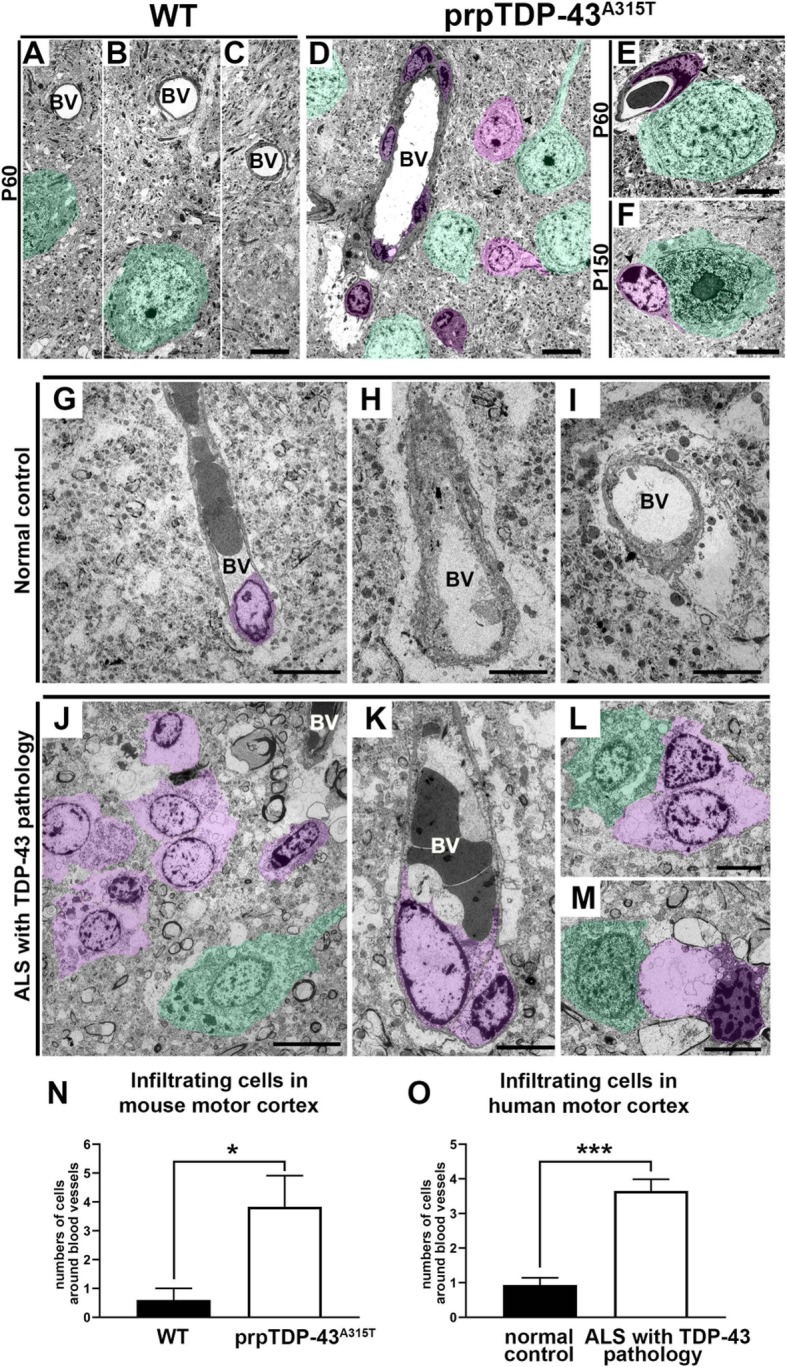
Non-neuronal glial cells are in close proximity to CSMN in the prpTDP-43^A315T^ mouse and to Betz cells in ALS cases with TDP-43 pathology. **a–c** Representative electron microscopy (EM) images in the motor cortex of WT mice. **d** A low-magnification EM image of motor cortex from prpTDP-43^A315T^ mouse shows degenerating CSMN (green), several non-neuronal glial cells within blood vessels (dark purple) and in the brain parenchyma (light purple). **e**, **f** Representative high-magnification EM images of glial/macrophagic cells (purple) adjacent to CSMN. **g**–**i** Representative EM images of the primary motor cortex (Brodmann area 4) isolated from normal controls showing blood vessels with few non-neuronal cells (dark purple). **j** A low-magnification EM image of the primary motor cortex (Brodmann area 4) isolated from ALS cases with TDP-43 pathology shows a degenerating Betz cell (green) with several non-neuronal glial cells in the vicinity (light purple). **k** A representative EM image of a blood vessel in motor cortex of ALS cases with TDP-43 pathology showing glial/macrophage protruding out (light purple). **l**, **m** Representative EM images showing degenerating Betz cells (green) in close proximity to glial/macrophages (light purple). **n** Graph represents average number of infiltrating cells around blood vessel per section in the mouse motor cortex of WT (black column) and prpTDP-43^A315T^ (white column). **o** Graph represents average number of infiltrating cells around blood vessel per section in the human motor cortex of normal controls (black column) and ALS with TDP-43 pathology (white column). BV, blood vessel. *p < 0.05, ***p < 0.0001. Scale bar: **a**–**f**, **h**, **i**, **k**–**m** = 5 μm. **f**, **i** = 10 μm

We next focused our attention to the brain parenchyma and the blood vessels, taking advantage of high-resolution EM. In the WT motor cortex, the blood vessels were lined with a thin layer of epithelial cells, and no infiltrating cells from the blood vessel were observed (Fig. 6a–c). In contrast, numerous non-neuronal cells were infiltrating from the blood vessels of prpTDP-43^A315T^ mice (Fig. 6d–f) even at P60 (WT: 1 ± 1 cell, *n* = 5 blood vessels, *n* = 3 mice; prpTDP-43^A315T^: 4 ± 1 cell, *n* = 6 blood vessels, *n* = 3 mice, *p* = 0.02; Fig. 6n). The blood vessels were enlarged, and the epithelial lining harbored many infiltrating monocytes that moved towards the brain parenchyma. These were cells of phagocytic origin. Their lumen was white, including large lysosomes, and their nucleus revealed their cellular identity to be a monocyte and macrophage lineage [19]. Interestingly, these infiltrating cells were mostly present in close proximity to large CSMN in the motor cortex, and some of them were found not only having contact but intruding into the CSMN soma, pushing the cytoplasm and even the nucleus. Some even broke the cell membrane and were engulfing parts of the CSMN cytoplasm as early as P60.

### Infiltrating monocyte invasion is confirmed in ALS with TDP-43 pathology

We next investigated whether Betz cells of patients with TDP43 pathology were subject to infiltration of the peripheral immune cells. In the motor cortex of normal controls, blood vessels were not enlarged, and they lacked infiltrating cells (Fig. 6g–i). Interestingly, similar to CSMN of prpTDP-43^A315T^, Betz cells of patients were also surrounded by neuroimmune cells (Fig. 6g–m, o), and blood vessels of patients with TDP-43 pathology were enlarged and many neuroimmune cells with large soma and nucleus were breaking off from the epithelial lining to enter the brain parenchyma, moving towards the diseased Betz cells (Fig. 6j). A diseased Betz cell was associated with at least one cell that is filled with lysosomes, and at times more than one neuroimmune cell was attached to a single Betz cell (patients with TDP-43 pathology: 4 ± 1 cell, *n* = 23 blood vessels, *n* = 4 subjects, *p* < 0.0001; Fig. 6l, m, o). However, Betz cells of control cases were not associated with neuroimmune cells (normal control: 1 ± 1 cell, *n* = 15 blood vessels, *n* = 3 subject) (Fig. 6o). The human post-mortem samples represent the end-stage of the disease when infiltrating monocytes and neuroimmune cells are most apparent. However, it is important to note that as observed in Figs. 5 and 6a–e, by P60, there are already infiltrating immune cells moving towards the diseased CSMN and are engaging in cell-cell contact with them, suggesting that the “call” has been made early during disease progression.

**Fig. 7:**
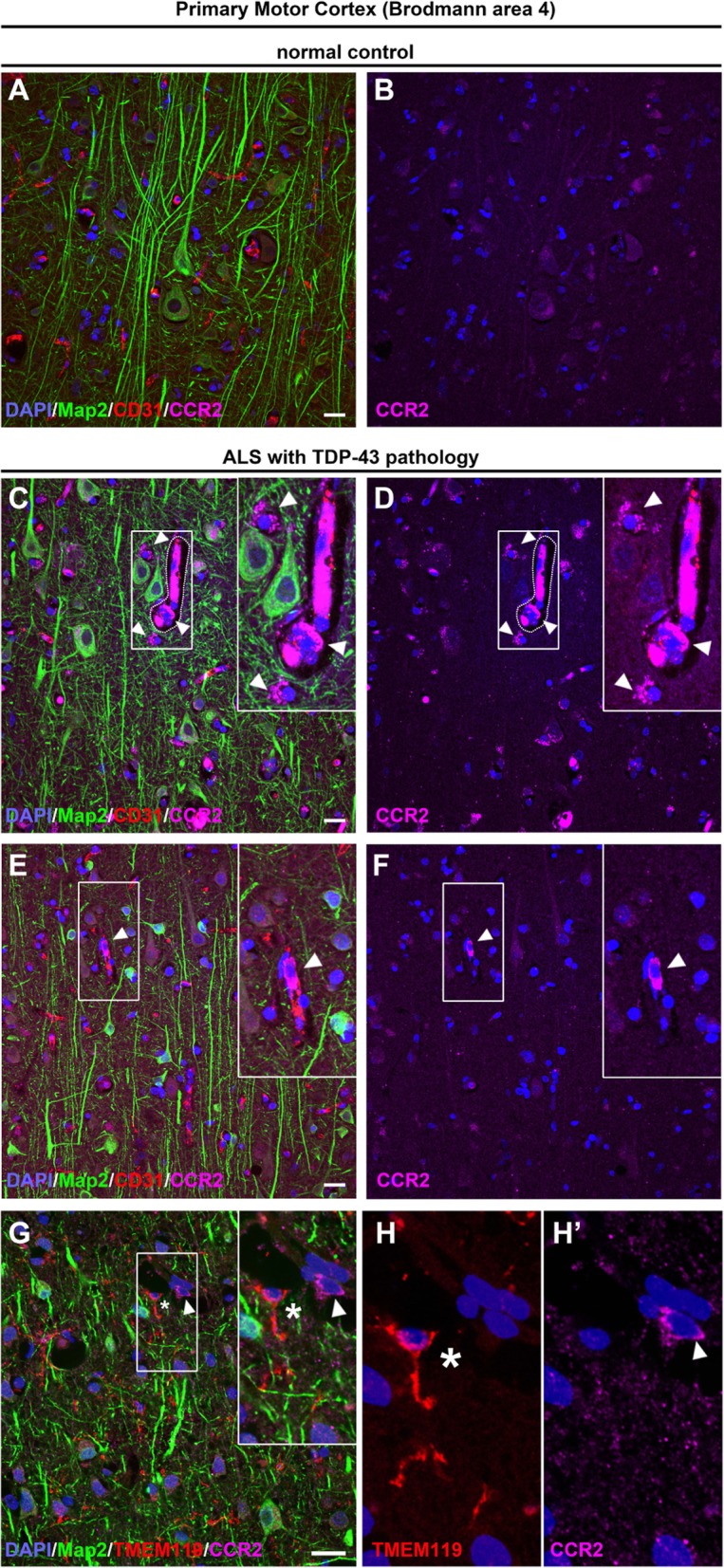
Evidence of infiltrating monocyte contribution in motor cortex of patients with TDP-43 pathology. Images of layer 5 of the primary motor cortex (Brodmann area 4) in normal controls and postmortem ALS cases. **a**, **b** Betz cells look healthy with normal apical dendrites in normal controls, and infiltrating monocytes (CCR2+) are not detected. **c**–**f** Numerous CCR2+ infiltrating monocytes are present in the motor cortex of patients with TDP-43 pathology. **c**, **d** Infiltrating monocytes are found in close proximity to degenerating Betz cells and within or near blood vessels (arrowheads). Insets enlarged within the panel. **e**, **f** A representative image showing an infiltrating monocyte in the brain parenchyma (arrowheads). Insets enlarged within the panel. **g**, **h** A representative image of motor cortex from patients with TDP-43 pathology shows infiltrating monocytes (CCR2+; arrowhead) that lack TMEM119 expression (asterisk) within the blood vessel. Inset is enlarged to the right. Scale bar = 20 μm

CCR2 expression profile helped reveal the identity of infiltrating cells in the motor cortex of ALS patients to be an infiltrating monocyte. CCR2+ infiltrating monocytes were not detected in normal controls (Fig. 7A, B), but there was a dramatic increase in the levels of CCR2+ infiltrating monocytes in the primary motor cortex of ALS with TDP-43 pathology. In these cases, CCR2+ infiltrating monocytes were detected inside blood vessels and in their vicinity, indicating possible recent transvasation (Fig. 7C, D, arrowheads). CCR2+ infiltrating monocytes were mainly found in areas where degenerating apical dendrites of Betz cells were present in the brain parenchyma (Fig. 7E, F). Similar to the results obtained in the mouse motor cortex, lack of TMEM119 expression further revealed that CCR2+ cells were infiltrating monocytes and not of microglia origin in the motor cortex of human patients (Fig. 7G, H).

### MCP1 expression increases in the Betz cells with ALS with TDP-43 pathology

**Fig. 8:**
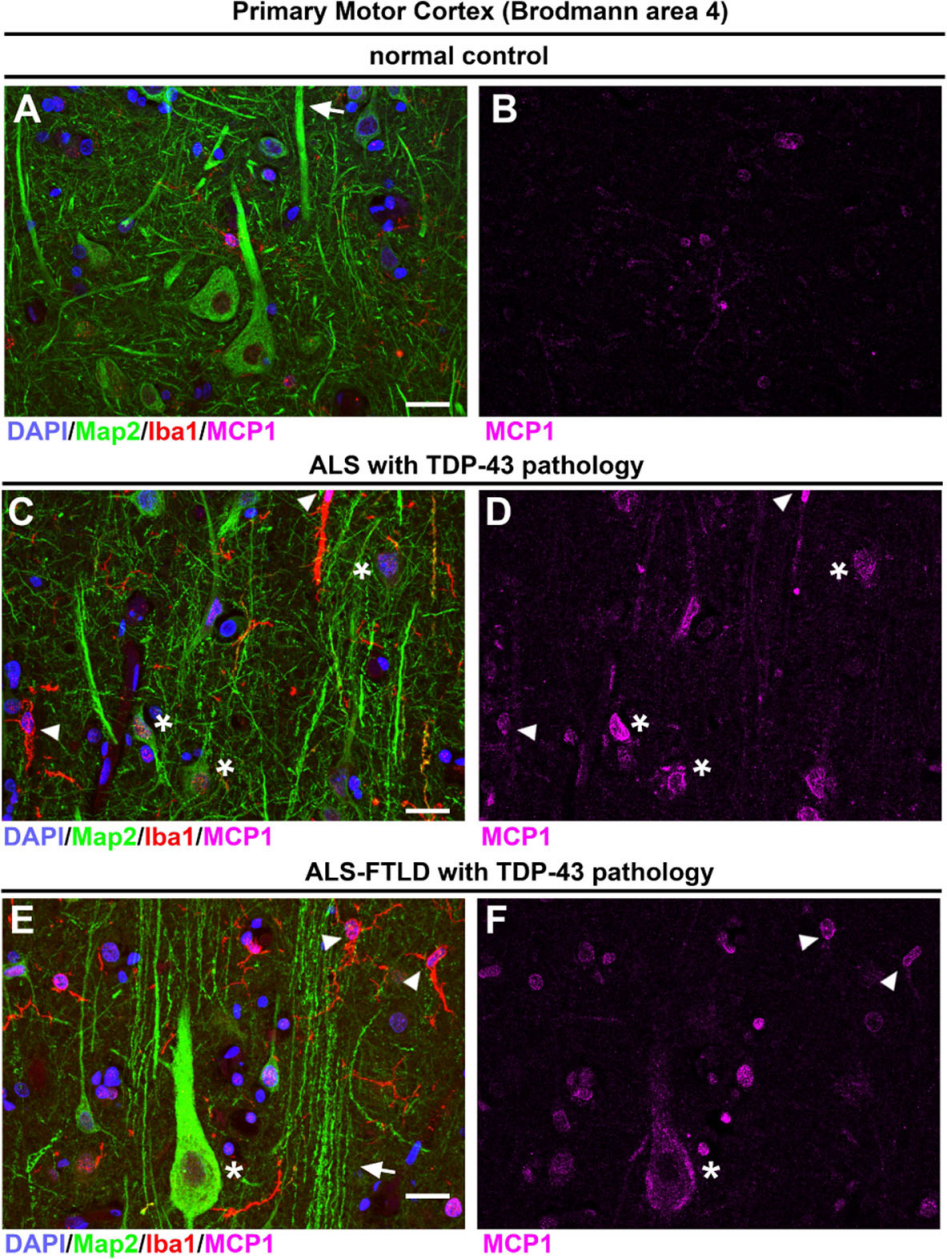
Neuronal expression of MCP1 increases in ALS cases with TDP-43 and ALS-FTLD cases with TDP-43 pathology. Images of layer 5 of the primary motor cortex (Brodmann area 4) isolated from normal controls and postmortem ALS cases. **a**, **b** Normal controls express low levels of MCP1 and Betz cells (Map 2+) have healthy apical dendrites (arrows). **c**, **d** ALS cases with TDP-43 pathology have smaller Betz cells or degenerating Betz cells (asterisks) that express MCP1 and they are surrounded by activated microglia (Iba1+, arrowhead). **e**, **f** ALS-FTLD with TDP-43 pathology cases have Betz cells expressing high levels of MCP1 (asterisk), and there are numerous degenerating apical dendrites (arrows) with activated microglia (arrowheads) near degenerating apical dendrites. Scale bar = 20 μm

The presence of CCR2+ infiltrating monocytes in the motor cortex of ALS cases with TDP-43 pathology prompted us to investigate whether Betz cells express the chemokine MCP1 [10, 21]. Betz cells with healthy apical dendrites have either no or very low levels of MCP1 throughout the primary motor cortex in normal controls (5 ± 4%, *n* = 43 cells, *n* = 3 normal controls; Fig. 8a, b). In contrast, MCP1 expression was dramatically increased in both ALS cases with TDP-43 pathology (93 ± 7%, *n* = 72 cells, *n* = 4 patients, *p* < 0.0002; Fig. 8c, d) and ALS-FTLD cases with TDP-43 pathology (77 ± 4%, *n* = 44 cells, *n* = 3 patients, *p* < 0.002; Fig. 8e, f). Activated microglia also expressed high levels of MCP1, as we have previously shown in ALS pathology (arrowheads) [10]. These activated microglia surrounded degenerating Betz cells expressing high levels of MCP1 (asterisks). Interestingly, some large Betz cells currently with no sign of prominent degradation also displayed high levels of MCP1 (Fig. 8e, f), suggesting that increased MCP1 expression occurred prior to cellular engagement with CCR2+ cells, neuronal degradation, and clearance in the motor cortex of patients with TDP-43 pathology.

## Discussion

Neuroinflammation in the spinal cord potentially contributes to disease pathology in both ALS patients and mouse models of ALS [1, 6–9, 18]. However, the role of neuroinflammation in the ALS motor cortex is beginning to emerge [10, 22, 23]. Cerebral microglial activation was assessed in vivo during disease progression in ALS patients [23], and a correlation between microglia activation and pathophysiological changes of Betz cells was reported [22]. Since current evidence reveals an early cortical dysfunction [10], which occurs before symptom onset in ALS patients, recently more emphasis has been given to motor cortex. Yet, our understanding of the events that occur and how they relate to upper motor neuron loss or dysfunction is not complete.

Here, we investigated the motor cortex of both ALS and ALS-FTLD patients with TDP-43 pathology, which is defined with the presence of protein accumulations that contain phosphorylated TDP-43 protein and is detected in more than 90% of all ALS cases. This marks TDP-43 as the most common pathology observed in the broadest spectrum of ALS patients, including sporadic, familial, and ALS with FTLD cases [24]. Interestingly, TDP-43 pathology is detected even in patients without mutations in the *TARDBP* gene or patients with numerous different mutations [25, 26], further emphasizing the broad relevance of TDP-43 for ALS and ALS-FTLD. Therefore, understanding the basis of neurodegeneration with respect to TDP-43 pathology is important, and revealing the potential involvement of neuroimmune regulation is required to build better treatment options.

The mouse model of FTLD overexpressing TDP-43 in the forebrain (CaMKII-TDP-43 Tg) pointed at the connection between TDP-43 inclusions and neurodegeneration, providing evidence of a mechanistic link between FTLD and ALS [27, 28]. Besides, different point mutations of the *TARDBP* gene resulted in TDP-43 pathological neuronal cytoplasmic inclusions (NCIs) present in both ALS and FTLD cases [29], and they share common underlying pathologies [30]. We thus investigated the motor cortex of both ALS and ALS-FTLD patients.

Increased neuroinflammation in affected brain areas of FTLD patients and increased levels of cytokines in their cerebrospinal fluid suggested contribution of neuroinflammatory cells to disease pathology [9]. Presence of neuroinflammation with a robust innate immune response reinforced the idea of its contribution to pathogenesis in ALS and ALS-FTLD [9]. Therefore, we reasoned to investigate the motor cortex of ALS patients with TDP-43 pathology and utilize a mouse model of TDP-43 that closely mimics human condition. Among many different mouse models, we have selected the prpTDP-43^A135T^ mouse model as they displayed very prominent and progressive motor function defects with upper motor neuron loss [13]. We recently revealed the intracellular defects that occur in the Betz cells of ALS patients with TDP-43 pathology and how these cellular defects are closely recapitulated in the CSMN of prpTDP-43^A135T^ mice [14]. To bring cellular clarity to our analyses, we crossed prpTDP-43^A135T^ with UCHL1-eGFP mice [16] to generate prpTDP-43^A135T^-UeGFP mice, which have TDP-43 pathology and their CSMN are genetically labeled with stable and long-lasting eGFP expression [16].

The role of neuroinflammation in TDP-43 pathology was initially described in postmortem spinal cord with neuronal TDP-43+ inclusions associated with activated microglia [31]. Even though precise mechanisms of TDP-43 neurotoxicity remain unknown, TDP-43 disease models provide some clues and conflicting results [32]. For instance, inactivation of TDP-43 accompanied reactive astrogliosis and microglia activation and motor neuron loss in the spinal cord [33]. TDP-43 knockout mouse models also described gliosis in the ventral horn of the spinal cord with the accumulation of phosphorylated neurofilament in motor neurons [34]. Furthermore, the presence of TDP-43 pathology in astrocytes was capable of initiating neurodegeneration [35]. In contrast, a possible “neuroprotective” model of TDP-43 pathology was suggested in the rNLS8 mice, an inducible mouse model of ALS [36].

Previously, the presence of microgliosis in the motor cortex of the hSOD1^G93A^ ALS mouse model was reported [12]. A possible role of microgliosis in relation to CSMN degeneration became clear upon observation of cell-to-cell interactions between activated microglia and degenerating CSMN in the motor cortex of hSOD1^G93A^ mouse model [11]. Our study describes, for the first time, the presence of extensive astrogliosis and microgliosis in the motor cortex of ALS with TDP-43 pathology. The microglia activation patterns observed in the human brains are consistent with current microglia classification [18]. Moreover, we encountered unique rod-shaped microglia in all ALS patients and the prpTDP-43^A315T^ mouse model. These rod-shaped microglia were initially described by Franz Nissl more than 100 years ago [37], and their presence in the brain has been clinically associated with neurosyphilis, subacute sclerosing panencephalitis, lead encephalopathy, viral encephalitis including HIV-1, and Rasmussen’s encephalitis [37, 38]. Recent studies with experimental models have reported the formation of rod-shaped microglia in traumatic brain injury and focal transient ischemia, suggesting polarization of the microglia to follow along the injured neuronal processes [39, 40]. A recent publication demonstrated that rod-shaped microglia are uniquely present in the brains of patients with neurodegenerative diseases, including Alzheimer’s and Lewy body disease [41]. Interestingly, we detected the rod-like microglia especially along the degenerating apical dendrites of both Betz cells and CSMN, a phenomenon observed in both species, suggesting an important function that is conserved throughout evolution.

Our findings also reveal the presence of infiltrating monocytes, expressing CCR2 and lacking TMEM119 microglia marker expression, and display their cell-cell interaction with the Betz cells that express MCP1, a chemoattractant ligand for CCR2. These findings further suggest peripheral monocyte infiltration and the importance of MCP1-CCR2 interaction in the motor cortex of both ALS patients with TDP-43 pathology. Importantly, this finding is recapitulated in the motor cortex of the TDP-43 mouse model.

Increased expression of the chemokine MCP1 offers a possible mechanism for enhanced innate immune response and recruitment of peripheral Ly6C+ and CCR2+ infiltrating monocytes in ALS [42–45]. Such monocytes are prone to penetrate the brain parenchyma and acquire a macrocytic phenotype during disease [46]. Alterations in the peripheral immune response in ALS has been subject to many studies, and due to its dynamic nature, it is difficult to identify cells in action [21, 47, 48]. Here, we show proof for the involvement of infiltrating monocytes in the cerebral cortex and that the immune response includes both resident microglia and infiltrating monocytes. We detected increased astrogliosis and microgliosis especially in layer 5 and revealed the presence of infiltrating monocytes that penetrate the motor cortex. Since CSMN numbers were significantly reduced by P90 in the prpTDP-43^A315T^ mice [14], it is important to note that increased astrogliosis and microgliosis potentially contributed to UMN loss in the motor cortex.

Our findings demonstrate a close correlation between the cellular events that occur in the motor cortex of patients with TDP-43 pathology and a well-characterized mouse model of TDP-43. There is increased astrogliosis and microgliosis in the motor cortex, and rod-like microglia especially wrap around degenerating apical dendrites of the upper motor neurons. Infiltrating CCR2+ monocytes enter the brain parenchyma while diseased Betz cells express MCP1, further revealing the presence of MCP1-CCR2 signaling with respect to neuroinflammation and TDP-43 pathology. These findings show that ALS and ALS-FTLD patients share similar patterns of neuroinflammation and that this pattern is conserved among species.

Previously, cortical dysfunction was considered to be a byproduct of an ongoing degeneration, and thus the cortical component of the motor neuron circuitry was not considered to be a therapeutic target. However, developing evidence suggests the opposite. The upper motor neurons begin to degenerate very early in the disease [11, 14, 16]. The cortical dysfunction, mostly in the form of hyperexcitation, becomes evident even before symptom onset in ALS patients [4]. Targeting the health of motor cortex in the rat models of ALS improved not only the health of overall motor function but also the health of spinal motor neurons and even the integrity of the neuromuscular junctions [49]. Hence, the motor cortex is a viable target.

Therefore, we believe that our results reveal one of the critical components of neuroimmune modulation. Understanding its details allows development of effective therapeutic intervention strategies. For instance, blocking macrophage activation by NP001 showed promise in clinical trials in ALS patients with an inflammatory disease component [50]. Such studies can be improved when the mode of initiation and progression and the involvement of the neuroimmune axis is well characterized and when the cortical component of the motor neuron circuitry is also considered.

## Conclusions

Here, we show that MCP1-CCR2 axis is an important contributor to the neuroimmune reaction in the motor cortex of a broad spectrum of ALS with TDP-43 pathology. Further understanding of the cellular and molecular basis of neuroimmune dynamics will enable development of effective treatment strategies in ALS, ALS/FTLD and other related motor neuron diseases. Our findings set the stage for this quest.

## Data Availability

Not applicable.

## Abbreviations

ALS: Amyotrophic lateral sclerosis
CCR2: CC chemokine receptor 2
CSMN: Corticospinal motor neurons
eGFP: Enhanced green fluorescent protein
fALS: Familial ALS
MCP1: Monocyte chemoattractant protein-1
PFA: Paraformaldehyde
sALS: Sporadic ALS
UMN: Upper motor neuron
